# *In Vitro* and* In Vivo* Characterization of N-Acetyl-L-Cysteine Loaded Beta-Tricalcium Phosphate Scaffolds

**DOI:** 10.1155/2018/9457910

**Published:** 2018-07-31

**Authors:** Yong-Seok Jang, Phonelavanh Manivong, Yu-Kyoung Kim, Kyung-Seon Kim, Sook-Jeong Lee, Tae-Sung Bae, Min-Ho Lee

**Affiliations:** ^1^Department of Dental Biomaterials and Institute of Biodegradable Materials, Institute of Oral Bioscience and School of Dentistry (Plus BK21 Program), Chonbuk National University, Jeonju, Republic of Korea; ^2^Department of Dental Hygiene, Jeonju Kijeon College, Jeonju, Republic of Korea; ^3^Department of Bioactive Material Science, Chonbuk National University, Jeonju, Republic of Korea

## Abstract

Beta-tricalcium phosphate bioceramics are widely used as bone replacement scaffolds in bone tissue engineering. The purpose of this study is to develop beta-tricalcium phosphate scaffold with the optimum mechanical properties and porosity and to identify the effect of N-acetyl-L-cysteine loaded to beta-tricalcium phosphate scaffold on the enhancement of biocompatibility. The various interconnected porous scaffolds were fabricated using slurries containing various concentrations of beta-tricalcium phosphate and different coating times by replica method using polyurethane foam as a passing material. It was confirmed that the scaffold of 40 w/v% beta-tricalcium phosphate with three coating times had optimum microstructure and mechanical properties for bone tissue engineering application. The various concentration of N-acetyl-L-cysteine was loaded on 40 w/v% beta-tricalcium phosphate scaffold. Scaffold group loaded 5 mM N-acetyl-L-cysteine showed the best viability of MC3T3-E1 preosteoblastic cells in the water-soluble tetrazolium salt assay test.

## 1. Introduction

Bone tissue engineering is emerging as a significant potential alternative or complementary solution to repairing diseased or damaged tissue, enabling full recovery of the original state and function [1]. Calcium phosphate ceramics (CPCs) have been widely used as biomaterials for the regeneration of bone tissue because of their biocompatibility and ability to induce osteoblastic differentiation in progenitor cells [2, 3]. Among the CPCs, beta-tricalcium phosphate (*β*-TCP) bioceramics are widely used for hard tissue regeneration due to their excellent biocompatibility and their close similarity to biological apatite present in human bones [4, 5]. *β*-TCP is known to be highly resorbable* in vivo* with new bone ingrowths replacing the implanted *β*-TCP, which contributes significant advantage to *β*-TCP compared to unresorbable biomedical materials [4, 6]. In addition to the bioactivity and biocompatibility of the scaffold, the proper mechanical properties, scaffold pore morphology, porosity, and pore size are important to maintain the shape of scaffold against stress and for determining cell ingrowth [7, 8]. The mechanical strength of the scaffolds was usually low due to high porosity, large pore size, and interconnected structures. To overcome these limitations, a number of studies have been focused on improving mechanical strength of *β*-TCP bioceramics [9–11]. Several fabrication techniques, such as replication of polymer foams [12], freeze casting [13], gel-casting foaming [14, 15], and foaming with employment of several pore creating additives [16, 17], have been developed to control pore sizes, porosity, pores interconnectivity, and mechanical strength of *β*-TCP scaffold. Numbers of studies have been focused on the sintering properties of *β*-TCP bioceramics to improve their mechanical strength [18, 19]. Additionally, the scaffold structures must have sufficient porosity to allow for cellular infiltration and proper cell function. A porosity of 90% was recommended for optimum diffusive transport within a cell-scaffolds construct under* in vitro* conditions [20].

Supplemented osseointegration and shortened healing time are desired to guarantee a direct bone to implant adherence. Meanwhile, scaffolds employed for bone tissue regeneration should have highly porous structures with a well-interconnected 3D pore network to encourage cell growth, vascularization, and transport of nutrient and metabolic waste [21, 22]. Many pores enlarge to the surface and can be vascularized with an adequate diameter (>approx. 100 *μ*m) [23, 24]. Smaller pore diameters are more gainful in the adhesion and integration of mineralized tissues, contraction of the cell to implant, and the absorption of extracellular liquids [25, 26].

The wound infection is one of extremely severe issues in tissue regeneration and biomaterial implantation. An acute wound arising from a cut, surgical procedure, provides an opportunity for such bacteria to attack and colonize the underlying tissue, inclusive of connective tissue, muscle, and bone tissue. Bacterial contamination of implant results in tissue breakdown and degradation round the implant material. Recently, oxidative stress caused by excessive generation of intracellular reactive oxygen species (ROS) has been recommended to be associated with the adverse biological effects of such materials [27, 28]. N-acetyl-L-cysteine (NAC) is an antioxidant amino acid derivative and a sulfhydryl group, the functional half of NAC, directly resists ROS [29–33]. NAC can be harmonized into a cell and deacetylated into L-cysteine, a leader of glutathione [32, 34], which plays a key part in intracellular redox balance [35]. In fact, NAC prevented mastering of cell viability and function in fibroblasts and dental pulp cells created by resin [36, 37].

In this study, the effects of *β*-TCP contents and coating time on the mechanical properties and porosity of scaffolds developed by polyurethane replica method were investigated and then the effect of NAC loaded to *β*-TCP scaffold on biocompatibility was identified by various* in vitro* and* in vivo *tests.

## 2. Materials and Methods

### 2.1. Preparation of *β*-TCP Scaffolds

To fabricate interconnected porous *β*-TCP scaffold by the replicating, polyurethane (PU) foam (Joon Ang sponge, 45 PPI, Republic of Korea) cut off with dimension of 4 mm in height and width and 25 mm in length. This PU foam was cleaned in distilled water using ultrasonic cleaner (Branson 2210, USA) for 10 min and dried at 80°C for 24 h. The coating slurry was prepared using different concentrations (35, 40, 45, and 50 w/v%) of commercial *β*-TCP [Ca_3_(PO_4_)_2_, Sigma-Aldrich, Germany] powder, respectively. Briefly 5 g of polyvinyl butyral (PVB) (Sigma-Aldrich, USA) as binder was added in 100 ml of ethanol (C_2_H_6_O, Mallinckrodt Baker Inc, Malaysia) and vigorously stirred for 3 h, and then the given weight of *β*-TCP and 5 g of triphenyl phosphate (TEP) [(C_6_H_5_O)_3_PO, Sigma-Aldrich, USA] additive for increase in the fluidity of material were added. The mixture was again stirred for an additional 24 h.

The cleaned PU foam was immersed in the coating slurry for 1 min and then it was dried at 80°C for 5 min after blowing up by the compressed air through coated foam with airgun to disperse the slurry uniformly throughout the porous scaffolds without blocking the pores; dipping-and-drying steps were repeated twice and thrice in each concentration. Then the prepared samples were denoted as 35TCP2, 35TCP3, 40TCP2, 40TCP3, 45TCP2, 45TCP3, 50TCP2, and 50TCP3, respectively. The ceramic slurry coated foam was dried in drying oven at 60°C for 24 h. Then the prepared scaffolds were heat-treated to burn out the sponge and binder at 600°C for 3 h with the heating rate of 2°C/min and again solidified at 1300°C for 3 h with heating rate of 5°C /min.

### 2.2. The Scaffold Preparation for NAC Loading

The scaffolds were cleaned in 70% of ethanol three times and fully dried before loading of the NAC solution. Briefly, NAC (C_5_H_9_NO_3_S, Sigma-Aldrich, USA) solutions with different concentration (1, 5, 10, and 15 mM) were prepared with distilled water. NAC was loaded on each scaffold by dipping method and dried in vacuum oven at 37°C for 48 h, and the loading step was repeated five times.

### 2.3. Characterization of Scaffold

#### 2.3.1. Surface Morphology and Crystallinity

The surface morphology of the scaffolds was observed by scanning electron microscope (SEM, SUPRA 40 VP, Carl Zeiss, Germany). The average pore diameter of the scaffolds was calculated from SEM images by selecting five arbitrary areas. X-ray diffraction (XRD) was used to analyze the crystallographic structure and chemical composition of scaffolds after sintering using X-ray diffraction spectroscopic machine (Rigaku, Model D/mas 2550 v, USA) at 40 kV and 40 mA Cu K*α* monochromatic radiation from 2*θ* = 20° ~ 60° scanning angle with a step size of 0.02° and step scan rate of 1.8°/min.

#### 2.3.2. Total Porosity

The pores in the *β*-TCP scaffolds and the micro-tubes in the scaffold were responsible for the total porosity. The total porosity of the sintered *β*-TCP scaffold was determined by using the following equations [38]. Three identical specimens were used to determine the total porosity.

Total Porosity (%) = [1- (M / V × *ρ*)] × 100,

in which

  M is the mass of the sintered sample,
  V is the volume of the sintered sample,
 
*ρ* is the density of the sintered ceramic which is 3.07 g/cm^3^ [39].

#### 2.3.3. Mechanical Test

The flexural strength of the scaffolds was investigated using an Instron machine (Model: 4201, USA). Four-point bending test was performed with fabricated scaffolds of dimensions 3.5 × 3.5 × 28 mm^3^. The tests were carried out according to the procedure described in ASTM C1674-11, with the span in the bend test either 21 mm. The speed of the moving support was set at 0.126 mm/min in ambient conditions. Eight identical samples for each group were tested for each condition. The peak force recorded was used to calculate the flexural strength (S) from [40].

S = 3 PL / 4 bd^2^,

in which

  P is the break force,
  L is the outer (support) span,
  b is the specimen width,
  d is the specimen thickness.

### 2.4. Assessment of the Scaffold Bioactivity

*β*-TCP scaffold was immersed in simulated body fluid (SBF) solution at 37°C for 14 days; after that, it was distinguished for the formation of a calcium phosphate layer on the surfaces as one indication of bioactivity. The complement of the SBF was identical to that described by Kokubo et al. [41]. The scaffolds sizes of 8 × 8 × 4 mm^3^ were sterilized by soaking in 70% ethanol for 1 h and then left 30 min in UV light followed by being rinsed with phosphate buffered saline (PBS) three times and dried in clean bench for 24 h and then placed in falcon tubes containing 5 ml of SBF at 37 ± 2°C. The SBF was replaced every 24 h. After immersion test, the scaffolds were gently washed in distilled water and dried at room temperature for 24 h. Surface morphologies and chemical composition of byproducts formed on the surface were characterized by SEM, energy dispersive X-ray (EDX).

### 2.5. Cell Viability

#### 2.5.1. Cell Seeding and Culture

Mouse osteoblast cells (MC3T3-E1) were purchased from ATCC (American Type Culture Collection). 10% fetal bovine (FBS, Gibco Co., USA), 500 units/ml of penicillin (Gibco Co., USA), and 500 units/ml of streptomycin (Gibco Co., USA) were added to *α*-MEM (Gibco Co., USA) to prepare the culture medium. Incubation was carried out at 37°C in an atmosphere containing 5 vol. % CO_2_ according to ISO 10993-5:1999 [42]. The medium was replaced after 24 h then every 2 days. After the cells reached 80% confluency, the cells were harvested using a commonly procedure. The harvested cells were resuspended in the culture medium at a density of 2.4 × 10^4^ cells/ml.

Subsequently, the cell suspension was mixed with the medium and then dropped into the *β*-TCP scaffolds (1 ml/scaffold) and culture in 24-well culture plates, where *α*-MEM medium was used as the negative control; the cells were cultured in atmosphere at 37°C under a 5% CO_2_. The cell medium was changed every 2 days during the cell culture of 5 days. At the specified time endpoints, cell-scaffold media were removed and characterized for cell viability.

#### 2.5.2. Water-Soluble Tetrazolium Salt Assay Test

Cell viability was evaluated using the water-soluble tetrazolium salt (WST) assay (Fluka Co., Milwaukee, USA). The scaffold constructs were placed in 24-well plates with 1,000 *μ*l of cell medium for 5 days of culture. After culture time, the 100 *μ*l of media in each well was removed out, and then 100 *μ*l of WST solution was added newly for color expression. The scaffold samples were removed for absorbance evaluation. 200 *μ*l of supernatant from each well was transferred to a 96-well plate and the optical density (OD) measurements were conducted at a wavelength of 450 nm using a microplate spectrophotometer (EMax, Molecular Devices, USA). Six specimens for each group were tested, and each test was repeated three times.

### 2.6. Animal Experiment

We used skeletally two mature male beagles of which body weight was about 35 kg (HUVET, Icsan, South Korea). Housing and feeding of the animals were performed according to the NIH guidelines for the care and use of laboratory animals. The ethical approval of animal study was accepted by the Institutional Animal Care and Use Committee of the Chonbuk National University Laboratory Animal Center (CBNU 2017-0005).

Cylindrical scaffolds with 4 mm in diameter and 8 mm in length were made for* in vivo* test. For the implanted surgery, the beagle was premedicated with ketamine hydrochloride. General anesthesia of the animal was guided with thiopental and, after endotracheal intubation, maintained with halothane. Supplementary analgesia was obtained by local administration of 2% xylocaine containing epinephrine.

The left and right humeral and femoral diaphysis of animals were shaved and cleaned with ethanol-iodine and draped for sterile surgical procedure. The bones were exposed after transcutaneous incision and reflection of the periosteum. Eight corticocancellous defects (4 × 4 × 8 mm in height) at all the implant sites were prepared using slow drill speeds and plenty saline irrigation to minimize mechanical and thermal trauma to cortical bone. The final drill hole gave a slight press-fit of the transcortical implants which were inserted into the holes with 2 to 3 mm left proud of the periosteal surface. Two implants were carefully inserted into each humeral and femoral created defects of each animal side, and the 40TCP3 group in which NAC drug was not loaded was implanted into each femoral side for control group. 15mM40TCP3 group with the lowest cytotoxicity at* in vitro* was excluded for* in vivo* test.

After a healing period of 24 weeks, beagle was sacrificed. Immediately thereafter the implants were exposed and removed. After formalin fixation for 48 h, all bone samples were kept in 70% ethanol and scanned with micro-CT. The samples were thereafter stained in Villaneuva osteochrome bone stain (Polyscience, Inc.) and embedded in methyl methacrylate, monomer (Yakuri Pure Chemical So., LTD./Kyoto Japan). Histological analysis was performed to examine for new bone formation by optical microscopy (DM 2500M, Leica, Germany).

### 2.7. Statistical Analysis

All results of the control and experimental groups were analyzed independently. Statistical analysis was performed using Student's *t*-test at a significance level of *P* value < 0.05 to examine the difference in the variables among different experimental conditions.

## 3. Results

### 3.1. Characterization of *β*-TCP Scaffolds

#### 3.1.1. Microstructure and Crystallinity

**Figure 1:**
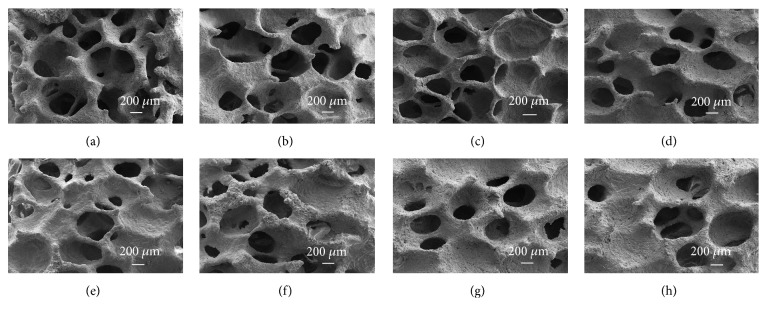
SEM morphologies of the *β*-TCP porous scaffolds with different concentration and coating times: (a) 35TCP2, (b) 35TCP3, (c) 40TCP2, (d) 40TCP3, (e) 45TCP2, (f) 45TCP3, (g) 50TCP2, and (h) 50TCP3. In the abbreviation, the number in front signifies the concentration (w/v%) of *β*-TCP powder included in slurry, and the number in back signifies the repetition number of immersion in slurry for fabrication of scaffold.

Figure 1 shows the typical SEM morphologies of the porous *β*-TCP scaffolds by varying the concentrations of *β*-TCP and coating times. When compared to the scaffolds at the same concentration, the stems of the scaffold became thicker and some pores were partly closed as the coating time increased. As the concentration of TCP increased, the stem became much thicker and some pores were completely clogged as shown in Figures 1(e)–1(h). Good porous structure with strong interconnected pores was observed in 40 TCP 2 (Figure 1(c)). It was revealed that *β*-TCP concentration and number of coating times visibly affected the interconnectivity and size of the pores.

**Figure 2:**
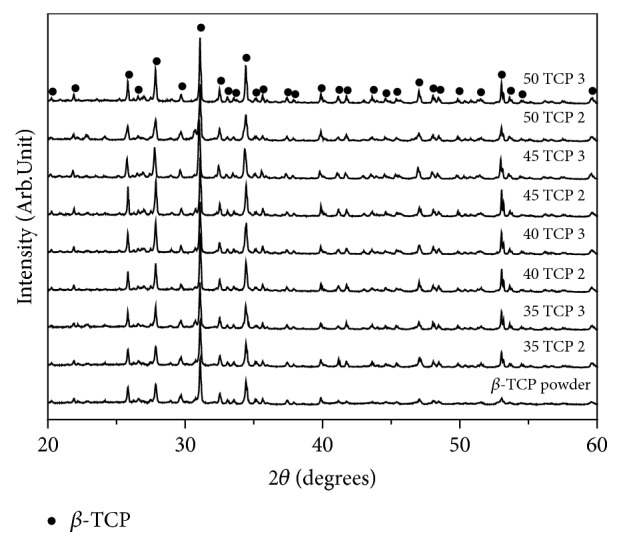
XRD pattern of the as-received *β*-TCP powder and scaffolds with different w/v% and coating times after sintering.

The XRD patterns of as-received *β*-TCP powders and sintered scaffolds prepared by different concentration of *β*-TCP and coating times were presented in Figure 2. The XRD pattern is composed of major phases of *β*-TCP (JCPDS # 09-0169), and it can be seen that the scaffolds sintered at 1300°C were mostly composed of highly crystalline and single phase *β*-TCP, and the intensity of *β*-TCP peaks was increased with increase in concentration and coating time.

#### 3.1.2. Porosity

**Figure 3:**
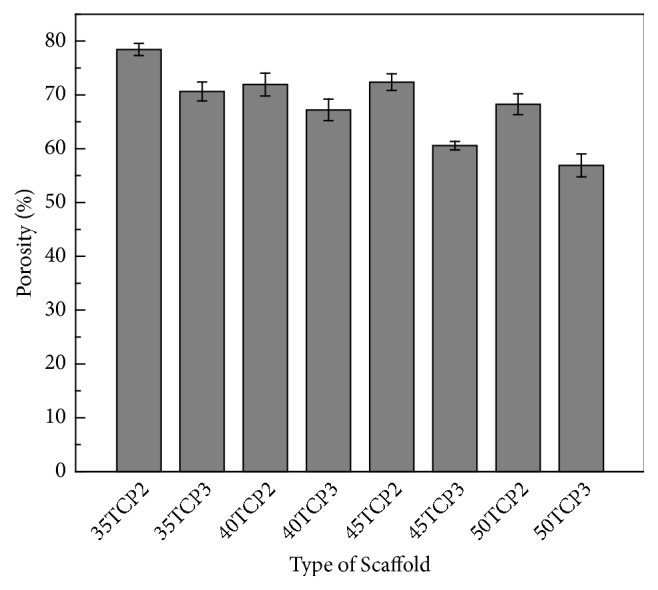
Porosity of *β*-TCP scaffold with TCP content and coating times. Error bar represents mean ± Standard Deviation (SD); *n* = 5.

Appropriate porosity and porous structure is important for cells penetration and ingrowth.  Figure 3 shows the total porosity of the scaffolds with different concentrations of TCP and coating times. The total porosity was decreased with increase in the concentrations of *β*-TCP and coating time.

#### 3.1.3. Mechanical Properties

**Figure 4:**
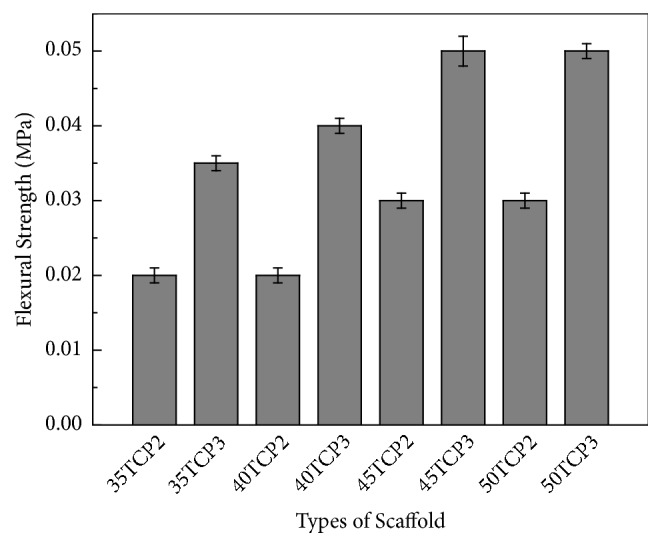
Flexural strength of *β*-TCP scaffold with concentration and coating times. Error bar represents mean ± Standard Deviation (SD); *n* = 5.

Figure 4 shows the influence of *β*-TCP concentrations and coatings time on the flexural strength of the sintered scaffolds. Increasing the concentrations and coating times, the flexural strength was increased. Results demonstrated that the flexural strength of the scaffold containing 35, 40, and 45 w/v%  *β*-TCP with three times coating was steadily increased while 50 w/v%  *β*-TCP showed the same strength as 45 w/v%  *β*-TCP.

### 3.2. *In Vitro *Bioactivity of NAC Loaded Scaffolds

#### 3.2.1. Immersion Test

**Figure 5:**
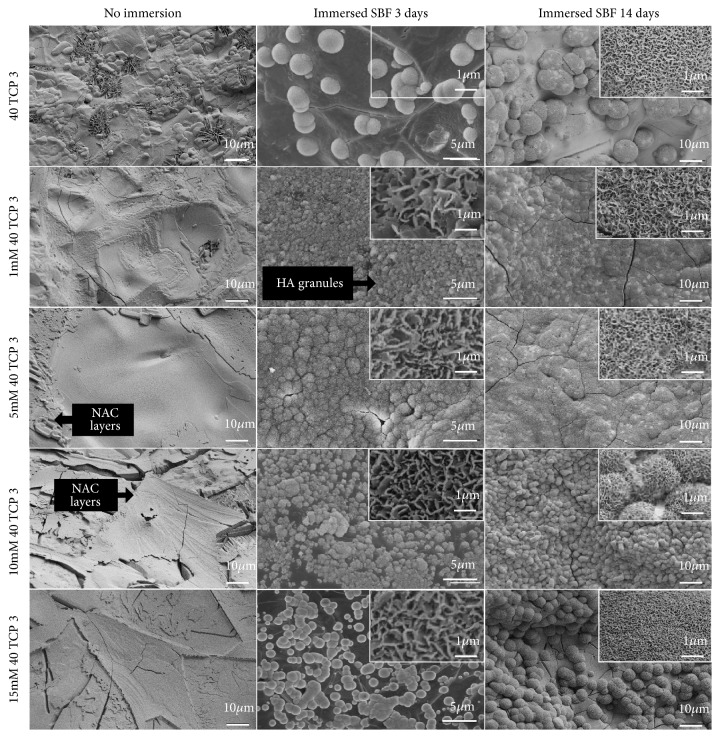
FE-SEM images of the surface of the *β*-TCP and NAC loaded scaffold before and after immersion for 3 days and 14 days in SBF.

Figure 5 represents surface morphologies of NAC loaded *β*-TCP scaffolds before and after immersion in SBF for 3 and 14 days. In comparison with the surface of 40 TCP 3, more and denser particulate layers were formed on the surface of NAC loaded 40 TCP 3 scaffolds within 3 days, and the densest particulate layer was observed on the surface of 5 mM 40 TCP 3 after immersion for 14 days. Porous network of needle-like crystals with nanometer size was identified in high-resolution FE-SEM images of all scaffolds after immersion.

Table 1 shows EDX results before and after immersion in SBF solution for 14 days. Compared with Ca/P ratio before immersion of 40 TCP 3, Ca/P ratio on 40 TCP 3 after immersion was gently increased, and the highest Ca/P ratio was confirmed on 10 mM 40TCP3, which was of 1.65.

#### 3.2.2. Cell Viability

**Figure 6:**
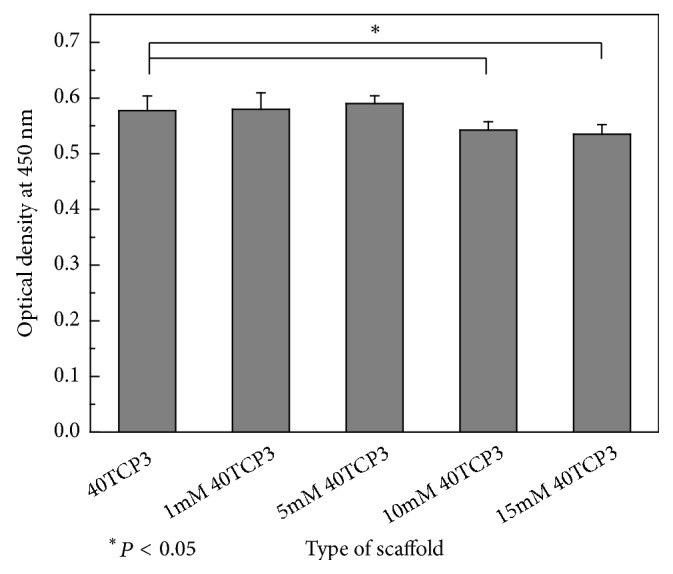
WST assay for viability of MC3T3-E1 at 5-day culture. Mean ± SD; *n* = 4. ^*∗*^*P* < 0.05 means significantly different.

Figure 6 presents the viability of MC3T3-E1 cells cultured for 5 days in the experimental groups to compare the effect of NAC loaded on *β*-TCP scaffolds on the cell proliferation. After 5 days of culture, higher cell growth was shown in the *β*-TCP scaffolds loading the lower concentration of NAC (below 5 mM) than 40TCP3 group (the control group). But in the groups loading NAC concentration over 10 mM, the cell viability showed a significant decrease (*p* < 0.05) compared to that in the control group.

### 3.3. Evaluation of Bone-Forming Effects

The surgical procedure and external radiation delivery were completed without major complications. Histological specimens were retrieved after 24 weeks following *β*-TCP scaffold implants, and tissue ingrowth to the periphery of the scaffold was evaluated.

**Figure 7:**
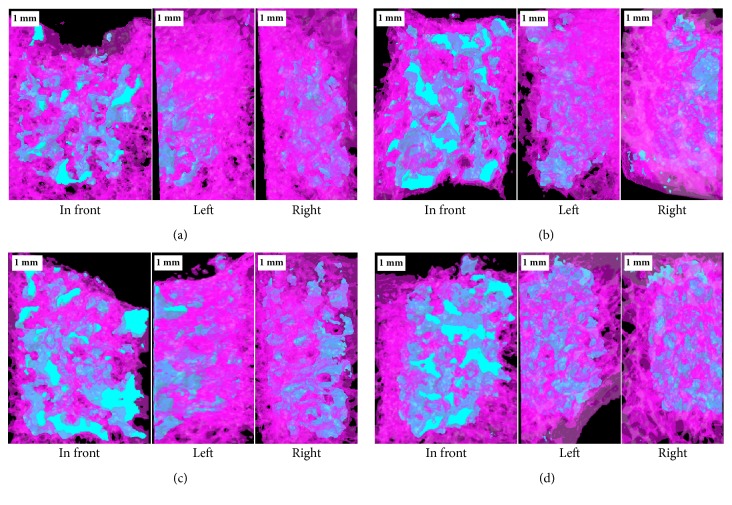
Micro-CT images at 24 weeks after implantation: (a) 40 TCP 3, (b) 1 mM 40 TCP 3, (c) 5 mM 40 TCP 3, and (d) 10 mM 40 TCP 3.

Figure 7 shows micro-CT images at 24 weeks after implantation of scaffolds, where the light blue in images is TCP blocks embedded in hole defect of bone. It was identified that the new bone penetrated into all porous TCP blocks and the degradation rate of 40 TCP 3 scaffold was decreased as the amount of NAC loading increased.

**Figure 8:**
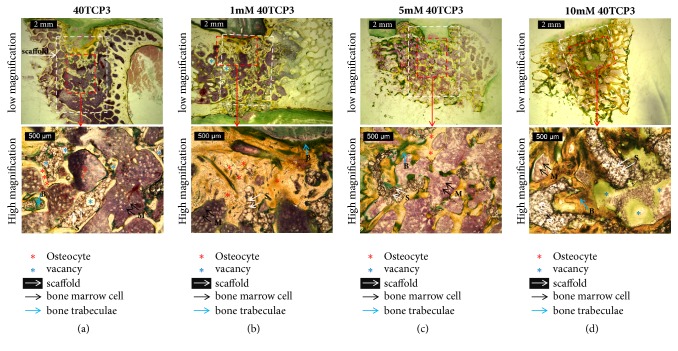
Histological analyses of humeral, femoral, and tibia bone by Villanueva Osteochrome bone staining at 24 weeks after implantation of each group: (a) 40 TCP 3, (b) 1 mM 40 TCP 3, (c) 5 mM 40 TCP 3, and (d) 10 mM 40 TCP 3. The number in front of 40 TCP 3 signifies the concentration of NAC in coating solution for loading NAC on the scaffold.

Figure 8 presents the histological results, where the positions of the scaffolds embedded in the cancellous bone are indicated by white squares. As the empty space of the bone was filled with scaffold, a new bone grew inside the scaffold from the boundary of the scaffold and bone. Defects in the compact bone at the upper part of the scaffold were seen in several groups and no inflammation was observed. The residual scaffold of the unloaded NAC (a) was observed to have many pores (blue *∗* marks) due to the porous form, and new bone cell was not formed inside the pores. In the group loaded with 1~5 mM NAC, dense binding of scaffold with new bone and bone marrow cell was observed. In the group loaded with 10 mM NAC(d), the pores of the scaffold were almost filled, so the new cartilage and bone marrow cells did not grow to the inside, and empty space was formed inner core.

## 4. Discussion

The objective of this study was to alter the pore structures of scaffolds for enhancing the mechanical properties with controlling the porosity and pore size by changing the concentrations of TCP slurry and number of coating times during the preparation.

Adequate porosity and mechanical strength of scaffolds are a prerequisite to allow its use in bone tissue engineering without rigid support. Pore size and porosity of scaffolds play a critical role in bone formation* in vitro *and* in vivo.* The porous structure of scaffold can provide good condition for cell growth and be easy to transport the nutrients and metabolic waste while the dense scaffold can impart excellent mechanical strength when implanted in body. The optimal pore size for bone tissue engineering required for bone ingrowth has been suggested in the range of 100-800 *μ*m [43]. Pores above 100 *μ*m allow to scaffold locating by osteoblasts [44]. And scaffolds must have sufficient porosity for nutrient and gas exchange. Previous research reported that a porosity of more than 80% was characteristic of an ideal scaffold [45, 46]. 40 TCP 3 scaffold had a pore size of 150-950 *μ*m and porosity of 67.22 ± 2% in this study. When compared to the prior study, it was similar to that observed on 13-93 bioactive glass with a pore size of 100-500 *μ*m and porosity was slightly different of 85 ± 2%, which was fabricated with a microstructure using a polymer foam replication technique [47].

The mechanical properties of the different concentration of *β*-TCP were examined by flexural strength measurements. As expected, the results indicated that the flexural strength of the 45 and 50 wt.% TCP scaffold was higher compared to that of 35 and 40 wt% TCP scaffold. Previous study also reported that the porosity would influence the mechanical properties of porous scaffolds [48]. Figure 4 shows that the flexural strength decreases progressively with the increase in porosity of the scaffolds. The mechanical properties of a scaffold used for tissue engineering are very important due to the need for the structural stability to resist the stresses that occurred during implantation* in vivo*. Even though 40TCP3 scaffold had a low flexural strength compared to a compressive strength in the range of 1 to 100 MPa of the cancellous bone [49], it had a good porous and interconnected pore in this study.

Mainly, TCP, whose calcium/phosphate (Ca/P) ratio is 1.5, has three crystal forms: *α*, *β*, and *γ*, which are thermodynamically determined by the sintering temperature of amorphous TCP. Despite a lower solubility than that of the *α*-form, *β*-TCP is markedly more soluble than synthetic HA that is verified by its solubility product constant (*α*-TCP: 3.16x10^−26^; *β*-TCP: 2.19x10^−30^: synthetic HA: 2.13x10^−59^). This suggests that *β*-TCP releases Ca cation (Ca^2+^) and P anion (P^i^), which play important role on bone homeostasis. Clearly, Ca^2+^ modulate osteoblastic viability, motility, proliferation, and differentiation through activation of calcium-sensing receptors, enhancement of Ca^2+^ influx into cells, and subsequent intracellular calcium signaling pathway [50, 51].

It was confirmed that application of NAC significantly alleviated cytotoxicity of *β*-TCP in the previous studies. A number of studies have indicated that NAC detoxifies oxidative stress-inducing cytotoxic material, particularly on the scaffold with NAC 1 mM and 5 mM loaded. Several presumable pathways of NAC-mediated detoxification had been presented. In the previous study, NAC alleviated intracellular level of ROS on/around *β*-TCP [31, 52]. Also, it was reported that NAC could directly displace extracellular ROS [30] and toxic compounds [53]. Thus, NAC encourages an increase in osteoblasts and reducing of inflammatory molecules [52, 54, 55]. We would need the further investigation for the most effective method to apply NAC on *β*-TCP scaffold material on the basis of the NAC-mediated detoxification mechanism and the physiochemical property of *β*-TCP scaffold. The results of this study suggested that application of antioxidant amino acid derivative NAC may offer a guide for the biofunctionalization of *β*-TCP scaffold-based material for bone regeneration. Likewise, this may provide important information for future development of synthetic bone substitutes.

Generally it has been known that the carbonated apatite is precipitated on the surface of biomaterial by immersion in a SBF. The precipitates are grown by extermination of the calcium, phosphate, carbonate, and hydroxide ions in the SBF [56]. The formation of a biologically active HA layer, which is chemically and structurally equivalent to the main mineral composition of bone, is a key requirement for developing a strong interfacial bond between bioactive ceramics and tissue* in vivo* [57]. The formation of HA surface layer* in vitro* is indicative of a material's bioactive potential* in vivo* [58]. In this study, the whole groups of the *β*-TCP scaffold illustrated that the formation of HA surface layer was observed within 3 days (Figure 6), which means that the *β*-TCP scaffold has the good bioactivity when considering that the formation of an amorphous calcium phosphate layer on the surface of 13-93 bioactive glass and the porous 45S5 glass-ceramic constructs was observed within 7 days and after 28 days in an SBF [47, 59]. More rapid formation of HA layer is adaptable for obtaining early biological fixation of a bioactive implant in bone repair and regeneration. The results provided useful information for designing the *β*-TCP scaffold with NAC of various concentrations. In this result, HA layer with Ca/P ratio of 1.65 was formed in 10 mM 40TCP3 group after immersion in SBF for 14 days, which was hightest compared with other gourps. Nevertheless, the estimation of the biocompatibility and bioactivity using* in vitro* test in SBF must be cogitated very carefully as it may differ from that of the practical* in vivo* reaction [60].

The MC3T3-E1 cells are a well-characterized mouse preosteoblastic cell line and have been used largely for* in vitro* cytotoxicity testing of biomaterials [61, 62]. As shown in Figure 6, good cell proliferation was identified in the *β*-TCP scaffolds loading the lower concentration of NAC below 5 mM, but less cell proliferation was confirmed in the *β*-TCP scaffolds loading the lower concentration of NAC over 10 mM than 40TCP3. It indicates that the proper amount of NAC loading on the *β*-TCP scaffold can increase biocompatibility of scaffold, but excess loading of NAC can cause cytotoxicity and reduce the site where the cells can attach and grow due to blocking the pores.

To investigate of the bone formation, histological samples were harvested 24 weeks after implantation of the *β*-TCP and NAC loaded scaffold. In all groups, empty defects of the bone were filled with the osteoblast and marrow cell based on the scaffold. The osteoblasts come from the differentiation of osteogenic cells in the tissue that covers the outside of the bone, or the periosteum and the bone marrow. The osteoblast is trapped inside of the bone after growth once it hardens, and it changed to be known as the osteocyte (the red *∗* marks) [63]. Through the loading of 1~5 mM of NAC, osteocyte areas including osteoblasts grew widely in healed defect areas. Furthermore, micro-CT 3D images and histological analysis revealed new bone formation and osteoblasts were plenty which occurred on the whole group of scaffold; at the same time the degradation of scaffold seemed slowly decreasing by increasing the ratio of NAC. The porous *β*-TCP scaffold served tissue ingrowth, indicating that connective tissue and bone-like tissue entered the block interior. The scaffold of the spongy structure plays a role of supporting the connection of the osteocytes, but the cells and the blood are hardly penetrated into the pores in the scaffold structure as 40TCP3 group. In previous study, it was reported that the percentage of viable osteoblasts was increased to 94% from 88% under *β*-TCP granules at 24 h by preaddition of NAC [64]. On the other hand, a high amount of NAC loading as 10 mM 40TCP3 in this study occurred to fill the outer pores of the scaffold, which make it difficult to grow inside of osteoblasts and circulate blood. The effect of scaffold pore size is the impact on bone regeneration and vascularization. The larger pore size around 350 *μ*m diameters can be partially responsible for the enhanced bone formation [65]. In this histological study, we found that the 1~5 mM 40TCP3 scaffolds elicit a higher level of osteoblastic activity, leading to higher mature bone formation.

## 5. Conclusions

This study focused on evaluating the biocompatibility of the NAC loaded *β*-TCP bone scaffold which was fabricated by replica method using slurries containing various concentrations of *β*-TCP and different coating times. The flexural strength of the interconnected *β*-TCP bone scaffolds was increased and its porosity was decreased as TCP content and coating times increased. Among the group, the porous 40TCP3 scaffold had the most appropriate interconnected porous structure and porosity. NAC loading within 10 mM on *β*-TCP scaffold significantly improved the osteoblastic response to the material. 15 mM NAC loaded *β*-TCP scaffold showed low cell viability and cell spreading on the surface of scaffolds. After implantation, the NAC loaded *β*-TCP scaffolds more effectively enhance the long-term bone remodeling and bone augmentation for bone formation than the scaffold without NAC loading* in vivo*.

## Figures and Tables

**Table 1:** Element composition of the scaffold after immersion in SBF for 14 days.

Sample abbreviation	Ca/P ratio	Chemical composition (at. %)		
		Ca	P	O
40TCP3 (Before immersion)	1.12	19.56	17.40	57.92
40TCP3	1.30	18.74	16.59	59.33
1 mM 40TCP3	1.35	19.59	14.55	58.34
5 mM 40TCP3	1.38	22.15	16.09	55.97
10 mM 40TCP3	1.65	25.65	16.10	53.52
15 mM 40TCP3	1.18	21.14	17.86	52.83

## References

[1] Hutmacher D. (2000). Scaffolds in tissue engineering bone and cartilage. *Biomaterials*.

[2] Cornell C. N. (1999). Osteoconductive materials and their role as substitutes for autogenous bone grafts. *Orthopedic Clinics of North America*.

[3] Samavedi S., Whittington A. R., Goldstein A. S. (2013). Calcium phosphate ceramics in bone tissue engineering: a review of properties and their influence on cell behavior. *Acta Biomaterialia*.

[4] Elliot J. (1994). *Structure and Chemistry of the Apatites and Other Calcium Phosphates*.

[5] Dorozhkin S. V. (2007). Calcium orthophosphates. *Journal of Materials Science*.

[6] Kim Y.-H., Jyoti M. A., Youn M.-H. (2010). In vitro and in vivo evaluation of a macro porous *β*-TCP granule-shaped bone substitute fabricated by the fibrous monolithic process. *Biomedical Materials*.

[7] Indolfi L., Baker A. B., Edelman E. R. (2012). The role of scaffold microarchitecture in engineering endothelial cell immunomodulation. *Biomaterials*.

[8] Hendriks J. A. A., Moroni L., Riesle J., de Wijn J. R., van Blitterswijk C. A. (2013). The effect of scaffold-cell entrapment capacity and physico-chemical properties on cartilage regeneration. *Biomaterials*.

[9] Perera F. H., Martínez-Vázquez F. J., Miranda P., Ortiz A. L., Pajares A. (2010). Clarifying the effect of sintering conditions on the microstructure and mechanical properties of *β*-tricalcium phosphate. *Ceramics International*.

[10] Ramay H. R. R., Zhang M. (2004). Biphasic calcium phosphate nanocomposite porous scaffolds for load-bearing bone tissue engineering. *Biomaterials*.

[11] Lin K., Chang J., Lu J., Wu W., Zeng Y. (2007). Properties of *β*-Ca3(PO4)2 bioceramics prepared using nano-size powders. *Ceramics International*.

[12] Sopyan I., Mel M., Ramesh S., Khalid K. A. (2007). Porous hydroxyapatite for artificial bone applications. *Science and Technology of Advanced Materials*.

[13] Lee E.-J., Koh Y.-H., Yoon B.-H., Kim H.-E., Kim H.-W. (2007). Highly porous hydroxyapatite bioceramics with interconnected pore channels using camphene-based freeze casting. *Materials Letters*.

[14] Ramay H. R., Zhang M. (2003). Preparation of porous hydroxyapatite scaffolds by combination of the gel-casting and polymer sponge methods. *Biomaterials*.

[15] Potoczek M., Zima A., Paszkiewicz Z., Ślósarczyk A. (2009). Manufacturing of highly porous calcium phosphate bioceramics via gel-casting using agarose. *Ceramics International*.

[16] Kundu B., Sanyal D., Basu D. (2013). Physiological and elastic properties of highly porous hydroxyapatite potential for integrated eye implants: Effects of SIRC and L-929 cell lines. *Ceramics International*.

[17] Descamps M., Duhoo T., Monchau F. (2008). Manufacture of macroporous *β*-tricalcium phosphate bioceramics. *Journal of the European Ceramic Society*.

[18] Li Y., Weng W., Tam K. C. (2007). Novel highly biodegradable biphasic tricalcium phosphates composed of *α*-tricalcium phosphate and *β*-tricalcium phosphate. *Acta Biomaterialia*.

[19] Laurent F., Bignon A., Goldnadel J. (2008). A new concept of gentamicin loaded HAP/TCP bone substitute for prophylactic action: In vitro release validation. *Journal of Materials Science: Materials in Medicine*.

[20] Freed L. E., Vunjak-Novakovic G., Biron R. J. (1994). Biodegradable polymer scaffolds for tissue engineering. *Bio/Technology*.

[21] Tampieri A., Celotti G., Szontagh F., Landi E. (1997). Sintering and characterization of HA and TCP bioceramics with control of their strength and phase purity. *Journal of Materials Science: Materials in Medicine*.

[22] Akao M., Aoki H., Kato K., Sato A. (1982). Dense polycrystalline *β*-tricalcium phosphate for prosthetic applications. *Journal of Materials Science*.

[23] Handschel J., Berr K., Depprich R. (2009). Compatibility of embryonic stem cells with biomaterials. *Journal of Biomaterials Applications*.

[24] Naujoks C., Langenbach F., Berr K. (2011). Biocompatibility of osteogenic predifferentiated human cord blood stem cells with biomaterials and the influence of the biomaterial on the process of differentiation. *Journal of Biomaterials Applications*.

[25] Eggli P. S., Muller W., Schenk R. K. (1988). Porous hydroxyapatite and tricalcium phosphate cylinders with two different pore size ranges implanted in the cancellous bone of rabbits. A comparative histomorphometric and histologic study of bone ingrowth and implant substitution. *Clinical Orthopaedics and Related Research*.

[26] Hing K. A., Annaz B., Saeed S., Revell P. A., Buckland T. (2005). Microporosity enhances bioactivity of synthetic bone graft substitutes. *Journal of Materials Science: Materials in Medicine*.

[27] Varma H. K., Sureshbabu S. (2001). Oriented growth of surface grains in sintered *β* tricalcium phosphate bioceramics. *Materials Letters*.

[28] Tsaryk R., Kalbacova M., Hempel U. (2007). Response of human endothelial cells to oxidative stress on Ti6Al4V alloy. *Biomaterials*.

[29] Yamada M., Ueno T., Minamikawa H. (2010). N-acetyl cysteine alleviates cytotoxicity of bone substitute. *Journal of Dental Research*.

[30] Yamada M., Ogawa T. (2009). Chemodynamics underlying N-acetyl cysteine-mediated bone cement monomer detoxification. *Acta Biomaterialia*.

[31] Spagnuolo G., D'Antò V., Cosentino C., Schmalz G., Schweikl H., Rengo S. (2006). Effect of N-acetyl-L-cysteine on ROS production and cell death caused by HEMA in human primary gingival fibroblasts. *Biomaterials*.

[32] Zafarullah M., Li W. Q., Sylvester J., Ahmad M. (2003). Molecular mechanisms of *N*-acetylcysteine actions. *Cellular and Molecular Life Sciences*.

[33] Schweikl H., Spagnuolo G., Schmalz G. (2006). Genetic and cellular toxicology of dental resin monomers. *Journal of Dental Research*.

[34] Tsukimura N., Yamada M., Aita H. (2009). N-acetyl cysteine (NAC)-mediated detoxification and functionalization of poly(methyl methacrylate) bone cement. *Biomaterials*.

[35] Kojima N., Yamada M., Paranjpe A. (2008). Restored viability and function of dental pulp cells on poly-methylmethacrylate (PMMA)-based dental resin supplemented with N-acetyl cysteine (NAC). *Dental Materials*.

[36] Yamada M., Kojima N., Paranjpe A. (2008). *N*-acetyl cysteine (NAC)-assisted detoxification of PMMA resin. *Journal of Dental Research*.

[37] Att W., Yamada M., Kojima N., Ogawa T. (2009). N-Acetyl cysteine prevents suppression of oral fibroblast function on poly(methylmethacrylate) resin. *Acta Biomaterialia*.

[38] Miao X., Tan D. M., Li J., Xiao Y., Crawford R. (2008). Mechanical and biological properties of hydroxyapatite/tricalcium phosphate scaffolds coated with poly(lactic-co-glycolic acid). *Acta Biomaterialia*.

[39] Narayan R., Colombo P., Widjaja S., Singh D. (2011). *Advances in Bioceramics and Porous Ceramics IV: Ceramic Engineering and Science Proceedings, Volume 32*.

[40] ASTM C1674-16 (2016). *Standard Test Method for Flexural Strength of Advanced Ceramics with Engineered Porosity (Honeycomb Cellular Channels) at Ambient Temperatures*.

[41] Kokubo T., Ito S., Huang Z. T. (1990). Ca, P-rich layer formed on high-strength bioactive glass-ceramic A-W. *Journal of Biomedical Materials Research Part B: Applied Biomaterials*.

[42] Biological evaluation of medical devices. Part 5. Tests for cytotoxicity: in vitro methods Arlington, VA: ANSI/AAMI ISO 10993-5. International organization for standardization.

[43] Sous M., Bareille R., Rouais F. (1998). Cellular biocompatibility and resistance to compression of macroporous *β*-tricalcium phosphate ceramics. *Biomaterials*.

[44] Kieswetter K., Schwartz Z., Hummert T. W. (1996). Surface roughness modulates the local production of growth factors and cytokines by osteoblast-like MG-63 cells. *Journal of Biomedical Materials Research Part B: Applied Biomaterials*.

[45] Attawia M. A., Herbert K. M., Uhrich K. E., Langer R., Laurencin C. T. (1999). Proliferation, morphology, and protein expression by osteoblasts cultured on poly(anhydride-co-imides). *Journal of Biomedical Materials Research Part B: Applied Biomaterials*.

[46] She Z., Zhang B., Jin C., Feng Q., Xu Y. (2008). Preparation and in vitro degradation of porous three-dimensional silk fibroin/chitosan scaffold. *Polymer Degradation and Stability*.

[47] Fu Q., Rahaman M. N., Sonny Bal B., Brown R. F., Day D. E. (2008). Mechanical and in vitro performance of 13–93 bioactive glass scaffolds prepared by a polymer foam replication technique. *Acta Biomaterialia*.

[48] Butler K. R., Benghuzzi H. A. (2003). Morphometric analysis of the hormomal effect on tissue-implant response associated with TCP bioceramic implants. *Biomedical Sciences Instrumentation*.

[49] Reilly D. T., Burstein A. H. (1975). The elastic and ultimate properties of compact bone tissue. *Journal of Biomechanics*.

[50] Blair H. C., Schlesinger P. H., Huang C. L., Zaidi M. (2007). Calcium signalling and calcium transport in bone disease.. *Subcellular Biochemistry*.

[51] Zayzafoon M. (2006). Calcium/calmodulin signaling controls osteoblast growth and differentiation. *Journal of Cellular Biochemistry*.

[52] Yamada M., Minamikawa H., Ueno T., Sakurai K., Ogawa T. (2012). N-acetyl cysteine improves affinity of beta-tricalcium phosphate granules for cultured osteoblast-like cells. *Journal of Biomaterials Applications*.

[53] Lee Y.-H., Lee N.-H., Bhattarai G. (2010). Enhancement of osteoblast biocompatibility on titanium surface with Terrein treatment. *Cell Biochemistry & Function*.

[54] Feng Y.-F., Wang L., Zhang Y. (2013). Effect of reactive oxygen species overproduction on osteogenesis of porous titanium implant in the present of diabetes mellitus. *Biomaterials*.

[55] Hanawa T., Kon M., Ukai H., Murakami K., Miyamoto Y., Asaoka K. (1997). Surface modification of titanium in calcium-ion-containing solutions. *Journal of Biomedical Materials Research Part B: Applied Biomaterials*.

[56] Hench L. L. (1998). Bioactive materials: The potential for tissue regeneration. *Journal of Biomedical Materials Research Part B: Applied Biomaterials*.

[57] Banerjee P., Irvine D. J., Mayes A. M., Griffith L. G. (2000). Polymer latexes for cell-resistant and cell-interactive surfaces. *Journal of Biomedical Materials Research Part B: Applied Biomaterials*.

[58] Chen Q. Z., Thompson I. D., Boccaccini A. R. (2006). 45S5 Bioglass^*Ⓡ*^-derived glass-ceramic scaffolds for bone tissue engineering. *Biomaterials*.

[59] Hirakata L. M., Kon M., Asaoka K. (2003). Evaluation of apatite ceramics containing *α*-tricalcium phosphate by immersion in simulated body fluid. *Bio-Medical Materials and Engineering*.

[60] Foppiano S., Marshall S. J., Marshall G. W., Saiz E., Tomsia A. P. (2004). The influence of novel bioactive glasses on in vitro osteoblast behavior. *Journal of Biomedical Materials Research Part A*.

[61] Inoue M., LeGeros R. Z., Inoue M. (2004). In vitro response of osteoblast-like and odontoblast-like cells to unsubstituted and substituted apatites. *Journal of Biomedical Materials Research Part A*.

[62] Ikeda Takeshi, Ikeda Kahori, Yamamoto Kouhei (2014). Fabrication and Characteristics of Chitosan Sponge as a Tissue Engineering Scaffold. *BioMed Research International*.

[63] Robert Dudley H., David S. (1961). The fine structure of bone cells. *The Journal of Cell Biology*.

[64] Galois L., Mainard D. (2004). Bone ingrowth into two porous ceramics with different pore sizes: An experimental study. *Acta Orthopædica Belgica*.

[65] Kuboki Y., Jin Q., Kikuchi M., Mamood J., Takita H. (2002). Geometry of artificial ECM: Sizes of pores controlling phenotype expression in BMP-induced osteogenesis and chondrogenesis. *Connective Tissue Research*.

